# p53: The Multifaceted Roles of Covalent Modifications in Cancer

**DOI:** 10.3390/ph17121682

**Published:** 2024-12-13

**Authors:** Tatiana A. Grigoreva, Angelina A. Romanova, Vyacheslav G. Tribulovich, Nikolay B. Pestov, Ruslan A. Oganov, Diana K. Kovaleva, Tatyana V. Korneenko, Nickolai A. Barlev

**Affiliations:** 1 St. Petersburg State Institute of Technology, St-Petersburg 190013, Russia; grigorevata@technolog.edu.ru (T.A.G.); romanova.aa@technolog.edu.ru (A.A.R.); tribulovich@technolog.edu.ru (V.G.T.); 2 Institute of Biomedical Chemistry, Moscow 119121, Russia; 3 Chumakov Federal Scientific Center for Research and Development of Immune-and-Biological Products, Moscow 108819, Russia; 4 Vavilov Institute of General Genetics, Moscow 119991, Russia; 5 Shemyakin-Ovchinnikov Institute of Bioorganic Chemistry, Moscow 117997, Russia; oganovra@my.msu.ru (R.A.O.); kovdinnk@gmail.com (D.K.K.); korn@mail.ibch.ru (T.V.K.); 6 Department of Biochemistry, Lomonosov Moscow State University, Moscow 19991, Russia; 7 Laboratory of Gene Expression Regulation, Institute of Cytology RAS, Saint-Petersburg 194064, Russia; 8 Department of Biomedicine, School of Medicine, Nazarbayev University, Astana 02000, Kazakhstan

**Keywords:** p53, posttranslational modifications, E3 ubiquitin ligases

## Abstract

The p53 protein has attracted huge research interest over several decades due to its role as one of the most important tumor suppressors in mammals, which orchestrates a synchronous response from normal cells in the body to various forms of stress. The diverse cellular activities of the p53 protein are regulated mainly via its post-translational modifications (PTMs). PTMs affect p53 on several levels: at the level of the assembly of tetrameric complexes on DNA to transactivate its target genes, at the level of the assembly of tetrameric complexes on DNA to transactivate its target genes; at the level of proteolysis in the absence of stress; and on the contrary, at the level of augmented protein stability in response to stress signals. Disruptions in these regulatory mechanisms can lead to deviations from normal cellular function, boosting tumor initiation and progression. Conversely, targeted interventions in these pathways could prove beneficial for the development of antitumor therapies. Advancing our understanding of p53 modifiers and the proteins involved in its regulation equips researchers with an expanded toolkit for studying cellular processes and for developing biologically active molecules that influence p53-mediated responses.

## 1. Introduction

The p53 protein, often referred to as the “guardian of the genome”, plays a crucial role in preserving DNA integrity in individual cells and preventing tumor formation in multicellular organisms. The p53’s essential functions in cancer prevention include regulating the cell cycle, activating DNA repair mechanisms, and initiating apoptosis whenever the accrued DNA damage is irreparable. The impairments of p53 function are strongly associated with the development of various cancers and their progression, underscoring its importance in oncology and cell biology.

Like other proteins, p53 undergoes numerous post-translational modifications (PTMs) that significantly influence its function and activity regulation. These modifications enable p53 to adapt to diverse physiological conditions, ensuring its activation in response to stress signals. Some general trends in these modifications are well known: for instance, phosphorylation enhances p53 transcriptional activity, while ubiquitinylation can promote its proteolysis. However, the complexity and variability of these modifications, coupled with the numerous proteins involved, necessitate a deeper understanding of the underlying processes.

Since the discovery of p53 and uncovering its role in the maintenance of genomic stability, the number of proteins identified as either its partners or its transcriptional targets has been steadily growing. Among these, the MDM2 ligase is of particular interest due to its critical role in ubiquitinylating p53, which in turn dramatically affects its functioning through the inhibition of p53 transactivation mechanisms, its nuclear export, and subsequent degradation. Inhibiting the interaction between p53 and MDM2 is considered a promising strategy in the development of anti-cancer therapies, and numerous research groups are actively pursuing advancements in this area [1,2,3,4]. Currently, more than 20 E3 ubiquitin ligases have been identified as regulators of p53, alongside numerous enzymes that mediate other PTMs and contribute to the assembly of active DNA-binding oligomers. Each of these post-translational enzymatic modifiers presents unique possibilities for targeted applications. Since PTMs enable p53 to adapt dynamically to changing conditions, fine-tuning these modifications can shift cellular processes toward either survival or apoptosis, influencing the fate of specific cells.

Understanding the mechanisms behind p53 activity and how PTMs regulate its function is essential not only for advancing cancer therapies but also for developing treatments for other conditions, including neurodegenerative and metabolic disorders. Here, we present an overview of the mechanisms governing p53 regulation at the protein level: from its oligomerization, which is essential for its transactivation function, to the ubiquitin-dependent proteolysis, which maintains low levels of this proapoptotic protein in normal cells. Our review highlights key enzymes involved in PTMs and the resulting effects on p53 function.

## 2. General Functions and Structure of the TP53 Gene and p53 Protein

**Figure 1:**
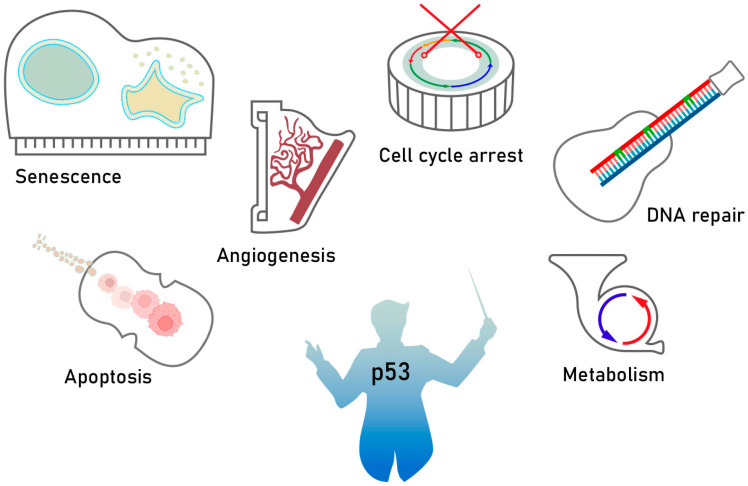
Different functions of the p53 protein in mammalian cells.

The p53 protein, encoded by the TP53 gene, is a crucial tumor suppressor in mammals. Acting as a transcription factor, p53 binds directly to DNA to exert its antitumor effects. This protein is essential for regulating various cellular processes, including apoptosis, cell cycle arrest, and DNA repair (Figure 1). Additionally, numerous studies have shown that p53 also plays a role in processes such as stem cell differentiation, autophagy, metabolic pathways, and ferroptosis [5,6,7].

Beyond its role in binding DNA, p53 also influences cell fate through direct interactions with other proteins. For instance, p53 can induce apoptosis by binding directly to anti-apoptotic proteins in the mitochondria [8]. In this interaction, p53 associates with anti-apoptotic members of the BCL-2 family (BCL-XL and BCL-2), which ordinarily bind pro-apoptotic proteins to stabilize the outer mitochondrial membrane.

Under normal conditions, p53 is continually degraded by the proteasome, and this degradation keeps its cellular levels and activity quite low. However, in response to cellular stress, p53 is stabilized through various PTMs, leading to its accumulation within the cell, where it activates its anti-oncogenic functions, potentially inducing apoptotic cell death. Indeed, the primary role of p53 is to support the normal function of individual cells in a multicellular organism, which includes preventing the transmission of damaged DNA during cell division and protecting against the emergence of abnormal cellular phenotypes.

**Figure 2:**
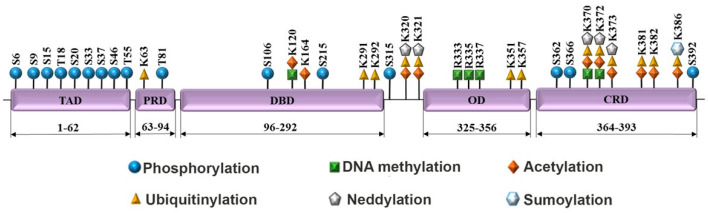
Major sites of post-translational modifications of p53 in the context of its domain structure. TAD, transactivation domain; PRD, proline-rich domain; DBD, DNA-binding domain; OD, oligomerization domain; SRD, C-terminal regulatory domain.

The TP53 gene consists of 11 exons, although two alternatively spliced exons (9β and 9γ) have been revealed in intron 9, thus making 13 potential exons in the gene. Nevertheless, in our review we will refer to the p53 gene with 11 exons [9]. The resulting protein contains 393 amino acid residues forming five main functional domains: the N-terminal transactivation, proline-rich, DNA-binding, oligomerization, and C-terminal regulatory domains. Each domain is a target for different PTMs that regulate the function and determine the fate of p53 (Figure 2).

The initiation of transcription from different promoters, alternative splicing of introns, the initiation of translation at different codons, and the presence of an internal ribosome entry site ensure the synthesis of numerous different variants of protein isoforms, in many of which the biological function is not known. The use of codons that initiate alternative translation from identical transcript variants results in extra isoforms [10]. In addition to canonical p53α (FLp53), mRNA transcripts encode 11 different isoforms [11], which differ from each other not just structurally—their functional properties are also quite different, and their assembly into hetero-oligomeric complexes greatly affects these properties. All isoforms modulate the activity of p53α and participate in the cellular response to stress by suppressing or enhancing the tumor suppressor activity of the canonical protein.

## 3. Oligomerization of p53

The formation of oligomers is essential for p53 to function effectively as a transcription factor, with tetrameric p53 demonstrating the highest DNA-binding efficiency, followed by dimeric complexes [12]. p53 oligomerization is facilitated by protein–protein interactions, regulated through PTMs, and is influenced by the cellular concentration of p53 [13]. Dimer formation occurs co-translationally on the polysome, whereas tetramer formation takes place post-translationally [14].

The formation of dimeric and tetrameric complexes of p53 molecules is facilitated by the presence of an oligomerization domain [15], and binding of other proteins to this domain can modulate oligomerization and affect p53 stability by promoting (MYBBPIA, BCCIP, 14-3-3, S100) or inhibiting (RBEL1A, ARC, S100A4, and S100B) the tetramerization process [13,16]). The PTMs of this site are also involved in the regulation of p53 oligomerization. For example, phosphorylation of p53 at S392 promotes tetramerization, and conversely, phosphorylation at S315 counteracts the stabilizing effect of phosphorylation at position S392 [16,17]. Nitration of p53 at Y327 has been shown to promote oligomerization and activation of p53 DNA-binding capacity, while oxidation at M340 destabilizes the p53 tetramer [16,17,18].

Both p53 dimers and tetramers can bind DNA in regulatory regions of their target genes; however, the dimer configuration enhances p53 DNA affinity approximately 160-fold, while the tetramer increases it more than a thousand-fold compared to the monomeric form [13,16]. Under normal conditions, p53 predominantly exists in a dimeric form, but in response to cellular stress, the proportion of tetramers rises significantly [13,19]. A key functional distinction is that only tetrameric p53 can induce apoptosis, as p53 dimers lack this capability [13].

The most active form of p53 is a homotetramer, comprising four identical p53 subunits [20]. Different isoforms and mutant forms of p53 can form unstable or weakly stable heterotetramers by combining various homodimers, often suppressing or altering p53’s transcriptional functions [17,21]). Mutant p53 forms (defective in oligomerization) compete with wild-type p53 for target DNA binding, thereby suppressing its canonical functions [21]. Heterotetramers that include both mutant and wild-type p53 exhibit lower DNA-binding affinity for target genes than p53 homotetramers and thus fail to achieve physiological levels of transactivation [22]. Furthermore, p53 isoforms that retain the oligomerization domain can associate with full-length p53α to form heterotetramers, which in turn reduces the homotetramerization and transcriptional activity of the full-length p53 protein [23,24].

In addition to the oligomers described above, p53 forms heterotetramers that include its homologs—the p63 and p73 proteins [25]. Homodimers of p63 and p73 can assemble into a p63/p73 heterotetramer, which is more thermodynamically stable than their homodimers; in contrast, the structural differences between the p53 and p63/p73 domains prevent their stable interaction [26]. The assembly of heterotetramers between oncogenic (mutant or isoforms of p53, ΔΝp63, and ΔΝp73) and anti-oncogenic members of the p53 family (p53wt, TAp63, and TAp73) correlates with the loss of oncosuppressor functions of p53 [27].

The exact molecular mechanism of p53 heterotetramerization is still debatable. However, the recent discovery that certain mutations in the DNA binding domain of p53 make the protein intrinsically unstable suggests that mutant p53 can form aggregates with WT p53, leading to its inactivation [28].

## 4. Post-Translational Modifications of p53 That Regulate Its Activity

The PTMs of p53 can be divided into two groups: those that affect protein activity (e.g., phosphorylation and acetylation) and those that modify p53 for subsequent degradation (ubiquitinylation, neddylation, and sumoylation). However, it should be noted that the two latter modifications arguably affect p53 protein stability indirectly, through subsequent ubiquitinylation and/or its export to the cytoplasm [29,30].

PTMs can occur at multiple sites on the p53 protein, with some amino acid residues capable of being modified by different chemical groups (Figure 2). The functional effects of these modifications depend not only on the specific site modified but are influenced by those at other sites of the same protein. Furthermore, the impact on p53—whether activating or suppressing its function—can vary significantly depending on the milieu of interacting proteins and a range of conditions, especially when the modification occurs at the same amino acid residue [31]. Our knowledge of p53 covalent modifications is far from complete due to scarce amounts of the protein and other technical limitations of mass spectrometry (e.g., low yields of peptides covalently modified on arginine and lysine residues after trypsin digest). For quite a while, we have been entertaining the hypothesis that histone modifications in the promoters of p53-dependent genes can provide guidance in the discovery of novel PTMs in p53 [32,33]. For example, the recent discovery of histone lactylation in macrophages [34] indicates that p53 can also undergo this modification (p53Kla) under hypoxic stress conditions [35]. In accordance with our hypothesis, p300, a member of the HAT family, was demonstrated to mediate the lactylation modification of not only H3 and H4, but also p53 [36], while the deacetylases HDAC1-3 and SIRT1-3 possess the de-L-lactylase enzyme activity [37]. Lactylation of p53 on K120 and K139 diminished its transcriptional activity. However, it should be noted that PTMs of p53 by histone-modifying enzymes do not always produce clear functional readouts. For example, G9a-mediated methylation of p53 on K373 is dispensable for its transcriptional activity [38].

Another example of a novel histone modification is acylation (propionylation and butyrylation) revealed recently in histone H4. H3K14 is the site of propionylation and butyrylation in vivo, and HAT and HDAC can catalyze the addition and removal of propionyl and butyryl groups [39].

### 4.1. Phosphorylation of p53

Phosphorylation was one of the first PTMs of p53 to be studied and is known to contribute to p53 stabilization and facilitate protein–protein interactions. In response to various cellular stresses, site-specific phosphorylation of p53 is triggered by several serine/threonine protein kinases, including ATM/ATR, CHK1/CHK2, DNA-PK, and BTK, among others [40,41].

Phosphorylation at residues S6, S9, S15, S20, S37, S106, and T18 activates p53-dependent mechanisms by disrupting interactions with the E3 ubiquitin ligase MDM2, which normally suppresses p53’s pro-apoptotic functions. Additionally, phosphorylation at sites such as S15, S33, S37, S46, S215, S392, and T81 modulates p53’s transactivation abilities by initiating the transcription of target genes [31]. Notably, S392 phosphorylation also promotes the mitochondrial translocation of p53, facilitating transcription-independent activation of apoptosis [31]. In contrast, phosphorylation at S315, S362, and S366 is tagged for ubiquitin ligase and promotes the ubiquitin-dependent proteasomal degradation of p53. Moreover, some amino acid residues of p53 undergo phosphorylation in the absence of stress. For example, in the case of T55, this contributes to the proteasomal degradation of p53, ensuring that the protein is maintained at low concentrations in the cell [31]. Interestingly, phosphorylation of p53 at T81 can also lead to the dimerization of p53 with p73 [31,42].

Dephosphorylation plays an important role in p53 regulation, since its prolonged activation in intact cells prevents it from restoring normal cell function. The dephosphorylation process is carried out by serine/threonine phosphatases PP1, PP2, and PPM [31].

### 4.2. Acetylation of p53

Acetylation is a key post-translational modification that regulates p53 DNA-binding and transcriptional activity while also inhibiting its interaction with the negative regulator MDM2. Consequently, the acetylation levels of p53 rise significantly in response to cellular stress, correlating with increased p53 activation and stabilization [43].

Six acetyltransferases, categorized into two groups, are involved in p53 acetylation: the first group, TIP60/MOF/MOZ, modifies p53 mainly in the DNA-binding domain (K120 and K164 lysine residues), whereas the p300/CBP/PCAF group mostly acts on the regulatory domain (K370, K372, K373, K381, K382, and K386) [41,43].

Disruptions to the acetylation of each site individually are compensated by modifications to other sites, while acetylation in all eight major sites completely inactivates the ability of p53 to transactivate CDKN1A (p21) and stops cell growth [43].

Acetylation at K120 promptly follows DNA damage, leading to the accumulation of acetylated p53 bound to the promoters of its pro-apoptotic transcriptional targets. The loss of K120 acetylation impairs the ability of p53 to induce the transcription of pro-apoptotic genes such as BAX and BBC3 (PUMA). Additionally, acetylation within the regulatory C-terminal domain promotes an open conformation of p53 by preventing the C-terminus from overlapping with the DNA-binding domain, thus enhancing p53 transactivation potential [41].

Conversely, deacetylation of p53 suppresses its transcriptional activity. HDAC1 mediates deacetylation at C-terminal residues K320, K373, and K382, while SIRT1 specifically targets K382 [41]. Together, acetylation and deacetylation enable the precise regulation of p53 in response to varying levels and types of cellular stress, thereby directing p53-dependent cellular outcomes, such as cell cycle arrest, apoptosis, or senescence.

Recently, acetylation of p53 at K120/164 by p300/CBP/Tip60 was reported to augment the expression of PD-1 [44]. Careful molecular analysis of p53 acetylation on these lysines made it possible to deduce that acetylation did not affect the affinity of p53 toward the PD-1 DNA response element per se. However, the recruitment of respective HATs (p300/CBP/Tip60) to the promoter region of the PD-1 gene enhanced its transcription. Furthermore, augmented expression of PD-1 attenuated the formation of tumors in nude mice. However, the authors found that, in response to γ-irradiation in mice, p53 activates the transcription of the PD-1 gene also in the developing thymus, potentially affecting the maturation process of cytotoxic T cells (CTLs). If true, this finding imposes an important ramification on the systemic use of p53-activating drugs as anti-cancer therapies. It is well established that high levels of PD-1 cause exhaustion and premature senescence of CTLs [45,46]. In this situation, such drugs would attenuate the anti-cancer immune response. In sum, this study raises the question of whether the systemic activation of p53 is always a good thing.

### 4.3. Methylation of p53

Methylation is another post-translational modification that modulates the protein–protein interactions and cell signaling pathways of p53. Methylation of p53 is carried out by a family of arginine methyltransferases (PRMTs) and lysine methyltransferases (KMTs) [47,48]. Lysine residues can be mono- (Kme1), di- (Kme2), or trimethylated (Kme3) at the ε-amino group [49].

Arginine methylation influences the specificity of p53 binding to a particular transcriptional target [50]. PRMT1 methylates p53 in the transactivation domain, whereas CARM1 appears to be specific to the C-terminal domain. These transferases act as coactivators of p53 and are involved in the methylation of histones H3 and H4, enhancing p53 transcriptional activity [48]. Using mass spectrometry, PRMT5 was found to methylate p53 at R333, R335, and R337, in response to etoposide treatment, suppressing p53 transactivation activity [50,51]. PRMT5 deficiency increases p53 binding to the promoters of apoptosis-inducing genes, leading to cell death, whereas PRMT5 overexpression induces cell cycle arrest through p21 activation.

Lysine methyltransferases such as SMYD2 and SET7/9 carry out C-terminal lysine modification of p53 and enhance or repress p53 transcriptional activity depending on the methylation site [47,48,52]. Interestingly, methylation precedes acetylation at neighboring positions and further promotes it [53]. SET7/9 enhances p53’s binding to promoters, facilitates the activation of target gene transcription, and inhibits SMYD2-mediated methylation at K372. Methylation of p53 lysine residues is believed to contribute to diverse biological effects, acting in conjunction with other PTMs. For example, demethylase KDM1 (LSD1) is responsible for p53 lysine demethylation [48], which reduces p53 interaction with its coactivator 53BP1 and subsequently inhibits apoptosis induction [54]. However, KDM1 is unable to demethylate monomethylated p53 at K370 or K372 [48].

To date, only one arginine demethylase, JMJD6, has been identified [51]. This oxygenase exhibits dual activity, catalyzing both lysine hydroxylation and arginine demethylation in histone and non-histone peptides [49]. JMJD6 is known to hydroxylate p53 at K382, counteracting acetylation mediated by p300/CBP [51,55]).

### 4.4. Ubiquitinylation of p53

Ubiquitinylation is a post-translational modification crucial for p53 intracellular localization and its protein stability [56]. For p53, several E3 ubiquitin ligases have been identified, categorized into four main types based on the structure of the domain that binds the E2 ubiquitin-conjugating enzyme: RING domain (MDM2, PIRH2, CARPs, synoviolin, TRIMs, MKRNs, RNFs, and TOPORS); HECT domain (ARF-BP1); U-Box domain (CHIP, UBE4B); F-Box domain (JFK) [57,58,59].

The E3 ubiquitin ligase ARF-BP1 includes a HECT domain in its structure and can directly bind and ubiquitinylate p53. The core tumor suppressor p14ARF, in turn, can bind both MDM2 and ARF-BP1, inhibiting their ubiquitin ligase activity [59,60]). Importantly, p14ARF can only inhibit MDM2-mediated polyubiquitinylation of p53, but not its monoubiquitinylation.

CHIP and UBE4B are examples of p53 ligases with a U-box domain. CHIP can polyubiquitinylate both wild-type and mutant p53 [57]. Notably, CHIP is highly expressed in adult human striated muscle, unlike other E3 ligases whose levels tend to remain constant or decrease with age, suggesting a role for CHIP in promoting p53 degradation in older or senescent cells [57].

In contrast to CHIP, UBE4B possesses both E3 and E4 ubiquitin ligase activities. UBE4B can directly interact with MDM2 to reduce p53 half-life and also regulates phosphorylated p53 at S15 and S392 within the nucleus, independently of MDM2 [61]. As an E4 ubiquitin ligase, UBE4B modulates ubiquitinylation levels by binding to ubiquitin or oligoubiquitin chains on substrate proteins, elongating and controlling chain length for further regulation [62]. Among the 68 known F-Box-containing proteins in humans, JFK is the only one containing a KELCH domain for protein substrate detection and ubiquitin residue attachment. JFK forms the SCF ubiquitin ligase complex that promotes the ubiquitinylation and proteasomal degradation of p53 [60]. As part of the complex, JFK is only able to recognize p53 phosphorylated at its DNA-binding domain [57].

The primary E3 ubiquitin ligase for p53 is MDM2, the best-known one in the class of RING-domain-containing ligases. The MDM2 gene is a transcriptional target of p53 and is activated in response to elevated p53 levels, maintaining stable p53 concentrations within the cell. MDM2 facilitates the transfer of activated ubiquitin from the E2 enzyme to the p53 protein, leading to p53 degradation via the 26S proteasome. MDM2 polyubiquitinylates p53 at several lysine residues in the C-terminal region [58,63]. Through this process, ubiquitin molecules are attached to p53 via isopeptide bonds between the C-terminus of ubiquitin and the ε-amino group of lysine residues on p53 [64]). Additionally, the N-terminal domain of MDM2 binds to the N-terminal domain of p53, inhibiting its transactivation capabilities and promoting the export of p53 from the nucleus to the cytoplasm.

Polyubiquitinylated p53, typically modified with four ubiquitin molecules, is directed to the 26S proteasome for degradation. Interestingly, genotoxic stress stabilizes p53 on the protein level not only by switching from its ubiquitinylation to acetylation but also by attenuating the activity of 26S proteasomes via phosphorylation [65]. In contrast, monoubiquitinylated p53 is transported from the cytoplasm to the mitochondria, where it interacts with BCL-2 family proteins. Under non-stress conditions, low levels of MDM2 primarily induce monoubiquitinylation of p53, a modification that is insufficient for its degradation [63]. When MDM2 binds to the transcription activator protein p300/CBP or KMT Set7/9, the ability of MDM2 to switch from mono-ubiquitinylation to polyubiquitinylation is attenuated [66].

MDMX (also known as MDM4) is a structural homolog of MDM2, though it lacks E3 ligase activity and cannot independently induce p53 degradation [5]. Similarly to MDM2, MDMX binds to the transactivation domain of p53, inhibiting its activity and forming the p53-MDM2-MDMX complex [67]. MDMX enhances MDM2’s E3 ubiquitin ligase function and stabilizes MDM2 by preventing its self-ubiquitinylation [67].

COP1 is another key negative regulator of p53, containing a RING domain that directs p53 for degradation [68]. In the absence of cellular stress, MDM2 and COP1 mutually enhance each other’s polyubiquitin ligase activity toward p53, maintaining its levels at a minimum [69].

PIRH2, encoded by the ZNF363 gene, binds and polyubiquitinylates only the tetrameric form of p53, suppressing its transactivation functions independently of MDM2 [70,71]. Like MDM2, ZNF363 is transcriptionally regulated by p53, forming a feedback loop. PIRH2 binds to the central DNA-binding domain of p53, but whether it can bind p53 simultaneously with MDM2 is unknown [71].

CARP1 and CARP2 also ubiquitinylate p53 for further degradation. Similarly to PIRH2, they can modify phosphorylated active p53 and thereby reduce p53 levels in the cell upon initiation of DNA damage repair [57,59]. Additionally, overexpressed SARPs can inhibit MDM2 self-ubiquitinylation by directly influencing its cellular levels [57]. The endoplasmic reticulum membrane protein synoviolin, which contains a RING domain, binds p53 in the cytoplasm and ubiquitinylates it, marking it for proteasomal degradation [60].

The TRIM family of proteins, comprising over 80 members with RING domains and E3 ubiquitin ligase activity, can ubiquitinylate p53 directly or by interacting with MDM2, targeting it for proteasomal degradation. Certain TRIM proteins (such as TRIM24 and TRIM32) are also involved in the p53 feedback loop [57,72].

The MKRN family of proteins (MKRN1 and MKRN2) polyubiquitinylates p53 [57]. MKRN1 modifies p53 by K291 and K292 for further degradation in the absence of stress, but in the case of genotoxic stress, it prefers p21 over p53 as the polyubiquitinylation substrate, and thus its activity favors the p53-dependent death of damaged cells [60].

The overexpressed E3 ubiquitin ligase RNF38 can promote the transport of p53 to distinct nuclear foci (PML nuclear bodies) as well as the ubiquitin-dependent degradation of p53 [73,74].

Another E3 ubiquitin ligase for p53 that contains a RING domain is TOPORS. Notably, beyond its role as a p53 inhibitor, this ligase can also sumoylate p53 when overexpressed, giving it a dual function similar to that of MDM2 [59].

E3 ligases can also mediate p53 ubiquitinylation without directing it to proteasomal degradation [59]. Depending on how ubiquitin binds to p53, this modification can result in nuclear export, activation, or inhibition of the transcription of p53 target genes [57]. In addition to MDM2, described previously, p53 nuclear export to the cytoplasm is facilitated by WWP1, which reduces p53 transactivation activity by directly binding to and mono-ubiquitinylating it [59,75]. Additionally, MSL2 independently ubiquitinylates p53 at residues K351 and K357, further promoting its translocation to the cytoplasm [57]. CUL7 also negatively impacts the transactivation activity of p53 [57]. Additionally, CUL7 has been shown to influence p53 oligomerization in the cytoplasm, reducing nuclear transport of the oligomerized p53 [59].

UBC13 facilitates the export of p53 from the nucleus to the cytoplasm, stabilizing it while reducing its transactivation abilities. UBC13 mono-ubiquitinylates p53 at K63, thus attenuating the MDM2-induced poly-ubiquitinylation of p53 [76]. Also, E4F1 oligoubiquitinylates p53 at residues K319-K321—distinct from the sites targeted by MDM2—promoting p53-dependent transcriptional activation of genes involved in cell growth arrest [77].

The extensive variety of E3 ubiquitin ligases targeting p53 enables the precise regulation of its activity and degradation, tailored to the specific tissues where p53 is expressed and across different stages of mammalian development [60]. Overexpression of p53-specific E3 ubiquitin ligases occurs in approximately half of all tumors, leading to the suppression of p53 antitumor functions and then to uncontrolled cell proliferation [78]. Consequently, the stabilization of p53 and activation of its tumor-suppressive functions through the inhibition of its interactions with E3 ligases—primarily MDM2—has become a pressing therapeutic goal [78,79,80].

### 4.5. Sumoylation and Neddylation of p53

Sumoylation and neddylation are modifications mediated by ubiquitin-like proteins, SUMO and NEDD8. Unlike ubiquitinylation, these modifications do not target p53 for proteasomal degradation; instead, SUMO and NEDD8 compete with ubiquitin for binding to lysine residues on p53 [57,76,81]. Ligases such as MDM2, FBXO11, PIAS, and TOPORS are known to mediate these modifications [57].

K386 of p53 is sumoylated by several SUMO isoforms (SUMO1, SUMO2/3) and mediated by members of the PIAS and TOPORS family [82,83]. Neddylation of p53 is mediated by MDM2 at residues K370, K372, and K373, and by FBXO11 at residues K320 and K321. Both sumoylation and neddylation are believed to inhibit p53 transactivation functions and, consequently, its tumor suppressor capabilities. However, the full effects of these PTMs on p53 remain poorly understood [76,83].

Interestingly, MDM2 is capable of both ubiquitinylating and neddylating p53. Moreover, the mechanism of selecting the induction of one or the other modification has not been investigated to date [83]. Also, neddylation of p53 is accomplished by the same lysine residues as acetylation. It is suggested that MDM2 and FBX011 compete with acetyltransferases for p53 modification at these sites [76].

## 5. Pharmacological Approaches Based on p53 Post-Translational Modifications

One of the traditional approaches to the restoration of the p53 function in malignant cells is the regulation of MDM2 ubiquitin ligase activity. This has been investigated for several decades, and various strategies have been employed to control p53 ubiquitinylation.

### 5.1. Peptide-Based MDM2 Inhibitors

Since p53 interacts with MDM2 through the α-helix, the general idea is to create a peptide that mimics the interaction motif. The peptides corresponding to the original p53 sequence motifs bind to MDM2 inefficiently due to the conformational problems of the protein–peptide interactions. To address this problem, cyclic helical peptides such as ATSP-7041 have been developed: these dual inhibitors of MDM2 and MDMX [84] efficiently activate the p53 pathway in tumors.

### 5.2. Small Molecules That Mimic Key Amino Acid Residues of p53

**Figure 3:**
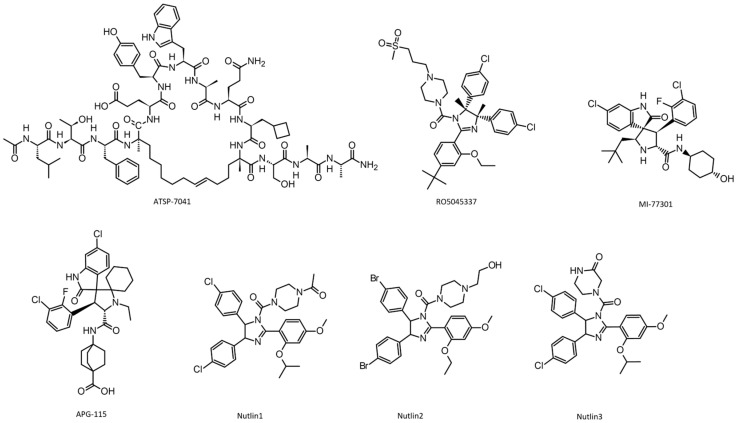
Molecular structure of effectors p53-MDM-2 interaction.

F19, W23, and L26 are the key residues in the p53 amino acid sequence involved in the interaction with MDM2. Nutlins (Figure 3) were the first potent and selective non-peptide small-molecule inhibitors of MDM2. Indeed, they were designed to inhibit the interaction between the MDM2 and p53, which promotes the degradation of p53. In cases where MDM2 is overexpressed (a common occurrence in various types of cancer), the function of p53 is significantly impaired. By blocking the MDM2-p53 interaction, nutlins stabilize p53, thus promoting cell cycle arrest, apoptosis (cell death), and the suppression of tumor cell growth [85].

More complex and biologically active compounds, such as RO5045337 (Roche), were developed, and a new generation of inhibitors based on the spiro-oxindole structure was born. For example, MI-77301 (Figure 3) demonstrated high binding efficiency to MDM2 but also rapid epimerization, which reduced its activity. Efforts to tackle this problem led to the discovery of APG-115 (Figure 3) with improved stability. Single-cyclic and polycyclic analogs of this drug are currently under development using various screening methods [86,87,88].

**Figure 4:**
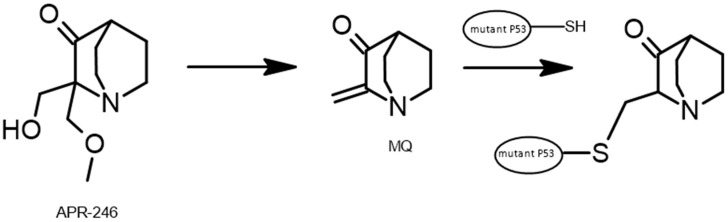
The conversion of APR-246 to the active substance.

APR-246 (Figure 4) is a pro-drug that, once inside living cells, converts into the active substance methylene quinuclidinone (MQ). MQ interacts with specific cysteine residues in the mutant p53 protein, restoring its functional state. Additionally, APR-246 can induce cell death independently of the presence of mutant p53 by affecting various cellular processes, such as maintaining redox homeostasis and ferroptosis, through the inhibition of enzymes like thioredoxin reductase [89].

Although the inhibitors of MDM2-p53 interaction effectively activate p53, their effects are limited to tumors with wild-type p53. Some may increase MDM2 levels, raising concerns about its p53-independent functions in the cell (for review see [67,90]).

### 5.3. Molecules That Directly Target MDM2

Direct negative regulation of MDM2 can activate p53. This can be achieved with small-molecule compounds of the PROTACs class (proteasomal targeting chimeras). Indeed, the rapid progress in bifunctional small molecules designed to induce interactions between target proteins and specific enzymes presents a nearly limitless potential—not only for targeted ubiquitinylation but also for phosphorylation, acetylation, and other modifications, provided research targets are carefully selected. The successful progression of several PROTACs through various stages of clinical trials, inducing the ubiquitinylation of clinically relevant targets, underscores the promising potential of this approach [56,91]. The PROTAC system consists of two “warheads” (ligands) cross-linked by a linker. One of the ligands specifically binds with a target protein and the other with the E3 ligase that directs the protein of interest (POI) to the ubiquitin–proteasome system. Some of the most common ligases for designing PROTACs are cereblon (CRBN) and Von Hippel–Lindau (VHL) E3 ligases with known ligands such as thalidomide, lenalidomide, pomalidomide, and the VHL ligand, respectively [92,93].

**Figure 5:**
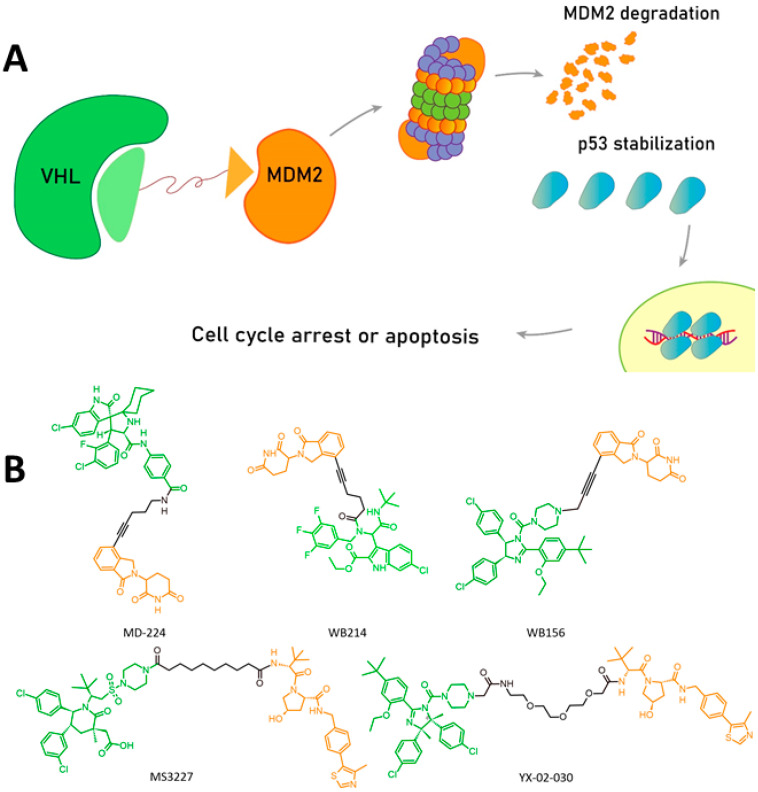
The mechanistic principle of PROTAC action and structures of PROTAC ligands. (**A**), PROTAC exemplified by a chimera of ligands for Von Hippel–Lindau (VHL) E3 ligase and MDM2, where VHL proximity leads to MDM2 degradation and p53 stabilization followed by tumor cell death. (**B**), typical PROTACs for p53 stabilization. POI ligands are shown in green, linkers in black, and E3 ligase ligands in orange.

In the case of MDM2 PROTACs, numerous disruptor variants have been developed based on specific MDM2 inhibitors that have already been characterized. For example, the MD-224 PROTAC consists of an MDM2 inhibitor like MI-1061 as a POI ligand and lenalidomide as the E3 ligand (Figure 5) [94]. In addition, the WB 156 compound that features nutlin as a POI ligand and lenalidomide as an E3 ligand led to MDM2 degradation and subsequent p53 activation, causing the high antitumor activity of the drugs [95].

Variation in linkers and ligands can alter the way the PROTACs work. For example, WD214 employs an MDM2-specific ligand that binds allosterically to p53, allowing for the destruction of both proteins [96]. Although PROTACS should be considered useful against cancers with mutant p53, another interesting compound, YX-02-030, holds promise against cancers irrespective of their p53 status, since it activates p73, a homolog of p53, which partially compensates for the loss of functional p53 [97]. At the moment, only a few PROTACs (like KT-253) have entered clinical trials (Table S1), whereas others currently are undergoing preclinical trials.

Aurora A kinase phosphorylates p53 at S315, promoting its ubiquitinylation by the MDM2 protein and subsequent proteolysis. This makes Aurora A a promising therapeutic target for p53 stabilization [98]. There are many Aurora A inhibitors (for review see [99]); a particularly interesting example is ENMD-2076b—it was shown that triple-negative breast cancer cells with mutated p53 and increased expression were more sensitive to the cytotoxic effects of ENMD-2076 than cells with reduced p53 expression [100].

## 6. Conclusions

It is essential to consider the complexity and multimodality of regulation when designing p53-activating therapeutics. Currently, the major efforts in modulating p53-mediated pathways are focused on the induction of apoptosis in tumor cells by preventing p53 proteasomal degradation. In this respect, PROTAC-driven therapeutics may constitute an interesting approach in p53-directed therapy. Toward this goal, inhibitors are being developed to disrupt the interaction between p53 and its principal E3 ubiquitin ligase, MDM2. However, despite many years of research and clinical trials, no clinically suitable candidate has yet been revealed. This is probably due to the underestimation of the complexity of the interactions mediated by p53. The fact that multiple p53 targets can play both positive and negative roles in the prevention of carcinogenesis complicates therapeutic strategies. Systemic administration of p53-activating drugs will likely suppress the anti-cancer immune response via enhanced expression of PD-1. Furthermore, active WT p53 was shown to confer survival benefits to cancer cells upon glucose [101], serine [102], or glutamine [103] starvation.

This overview provides a comprehensive understanding of cell-autonomous p53 regulation by PTMs. The remarkable complexity of p53 function as a tumor suppressor is influenced by a multitude of proteins through diverse PTMs that shape its conformation, stability, localization, and transactivation activity. The p53 protein is a crucial element in cellular regulation, promoting altruistic behavior in cells within multicellular organisms, including humans. The diversity of p53 PTMs allows for fine-tuning and quick adaptation of both normal and tumor cells to changing conditions. Studying the regulatory mechanisms of p53 not only deepens our understanding of fundamental cellular processes but also enables the development of targeted approaches for precise intervention in these pathways.

## Data Availability

No new data were created or analyzed in this study. Data sharing is not applicable to this article.

## Supplementary Materials

The following supporting information can be downloaded at: https://www.mdpi.com/article/10.3390/ph17121682/s1, Table S1: Recent clinical trials; Table S2: All clinical trials; Table S3: Referenced proteins.
